# Supplementary material for: Evaluating cognitive and affective abilities among medical students: behavioural and medicolegal perspectives
Source: PeerJ. 2024 Feb 2;12:e16864. doi: 10.7717/peerj.16864 (PMC10840496; doi:10.7717/peerj.16864)
Supplement: Supplemental Information 1 [file peerj-12-16864-s001.pdf]

Medicolegal Perspectives on Cognitive and Affective Abilities Among Medical Students.

this questionnaire aims to to evaluate and estimate the impact of exposure to medicolegal
issues on the cognitive and affective profile of the medical students

* Indicates required question

1. Do you agree to participate in the survey *

Mark only one oval.

- Yes
- No

2. Age *

3. sex *

Mark only one oval.

- Male
- Female

4. Original residence before university admission *

Mark only one oval.

- City
- Countryside

5. country *

Mark only one oval.

- Egypt
- Saudi Arabia
- Jordan
- Sudan
- America
- Canada
- Kuwait
- Tunisia
- Iraq
- Morocco
- Europe
- Others

6. College *

Mark only one oval.

- Nursing
- Pharamcy
- Physiotherapy
- Medicine
- Others

7. Level of education *

Mark only one oval.

- Undergraduate
- Postgraduate

8. Level for undergraduate (which year in the faculty?)

____

9. Specialty

Mark only one oval.

- Student
- Public health
- Forensic
- Histology
- Physiology
- Pharmacy
- Physiotherapy
- Nursing
- Surgery
- Nephrology
- Chest
- Obstetric
- Other: ________________________

10. Do you live with your family or in a different city? *

Mark only one oval.

- Family
- Another city

11. If you live in a different city?

Mark only one oval.

- Alone
- With friends

12. Do you have any chronic illness *

Mark only one oval.

- Yes
- No

13. Type of chronic disease *

Mark only one oval.

- Diabetes
- Hypertension
- Heart disease
- Liver disease
- Renal disease
- Others
- No

Cognitive Questionnaire

14. Difficulty in remembering details *

Mark only one oval.

- Rarely
- Sometimes
- Frequently

15. Difficulty in following directions (particularly oral) *

Mark only one oval.

- Rarely
- Sometimes
- Frequently

16. Easily distracted *

Mark only one oval.

- Rarely
- Sometimes
- Frequently

17. Does not remember what was just heard or read *

Mark only one oval.

- Rarely
- Sometimes
- Frequently

18. Difficulty in developing a timeline for assignments *

Mark only one oval.

- Rarely
- Sometimes
- Frequently

19. Difficulty in knowing where to start tasks *

Mark only one oval.

- Rarely
- Sometimes
- Frequently

20. Difficult time estimation/ judgment *

Mark only one oval.

- Rarely
- Sometimes
- Frequently

21. Has good ideas, but does not follow through *

Mark only one oval.

- Rarely
- Sometimes
- Frequently

22. Difficulty in organizing “stuff” *

Mark only one oval.

- Rarely
- Sometimes
- Frequently

23. Difficulty in expressing ideas *

Mark only one oval.

- Rarely
- Sometimes
- Frequently

24. Difficulty in maintaining a sequence *

Mark only one oval.

- Rarely
- Sometimes
- Frequently

25. Difficulty in problem solving *

Mark only one oval.

- Rarely
- Sometimes
- Frequently

26. Difficulty in considering another person’s perspective *

Mark only one oval.

- Rarely
- Sometimes
- Frequently

27. Difficulty in handling criticism *

Mark only one oval.

- Rarely
- Sometimes
- Frequently

28. Unaware of errors in work *

Mark only one oval.

- Rarely
- Sometimes
- Frequently

29. Difficulty in expressing myself when embarrassing situation? *

Mark only one oval.

- Rarely
- Sometimes
- Frequently

30. Overacting *

Mark only one oval.

- Rarely
- Sometimes
- Frequently

31. Difficulty in calming down and working *

Mark only one oval.

- Rarely
- Sometimes
- Frequently

32. Cries easily *

Mark only one oval.

- Rarely
- Sometimes
- Frequently

33. Frequent mood changes *

Mark only one oval.

- Rarely
- Sometimes
- Frequently

34. Low frustration tolerance *

Mark only one oval.

- Rarely
- Sometimes
- Frequently

35. Short attention span *

Mark only one oval.

- Rarely
- Sometimes
- Frequently

36. Overwhelmed by length/complexity of tasks to be done *

Mark only one oval.

- Rarely
- Sometimes
- Frequently

37. Rushing through work *

Mark only one oval.

- Rarely
- Sometimes
- Frequently

38. Difficulty in noticing details *

Mark only one oval.

- Rarely
- Sometimes
- Frequently

39. Forgets assignments or materials to complete them *

Mark only one oval.

- Rarely
- Sometimes
- Frequently

Part 2: Aggression measurement

40. Being provoked, I may react aggressively *

Mark only one oval.

- Yes
- No

41. If I have to resort to violence to protect my rights, I will. *

Mark only one oval.

- Yes
- No

42. I can think of hurting another person for no reason *

Mark only one oval.

- Yes
- No

43. I have threatened people I know *

Mark only one oval.

- Yes
- No

44. I have become so mad that I have broken things *

Mark only one oval.

- Yes
- No

45. I tell my friends openly when I disagree with them *

Mark only one oval.

- Yes
- No

46. I often find myself disagreeing with people. *

Mark only one oval.

- Yes
- No

47. When people annoy me, I may tell them what I think of them *

Mark only one oval.

- Yes
- No

48. I can’t stop arguments when people disagree with me *

Mark only one oval.

- Yes
- No

49. My friends say that I’m somewhat argumentative *

Mark only one oval.

- Yes
- No

Part 3: Personality assessment

50. Do you sometimes feel happy, sometimes depressed, without any apparent reason? *

Mark only one oval.

- Yes
- No

51. Do you prefer action to planning for action? *

Mark only one oval.

- Yes
- No

52. Do you have frequent ups and downs in mood, either with or without apparent cause? *

Mark only one oval.

- Yes
- No

53. Are you inclined to be moody? *

Mark only one oval.

- Yes
- No

54. Does your mind often wander while you are trying to concentrate? *

Mark only one oval.

- Yes
- No

55. Are you inclined to be quick and sure in your actions? *

Mark only one oval.

- Yes
- No

56. Are you frequently "lost in thought" even when supposed to be taking part in a conversation? *

Mark only one oval.

- Yes
- No

57. Do you feel motivated in some situations and indifferent in others? *

Mark only one oval.

- Yes
- No

58. Would you feel upset when you fail to make multiple social relationship? *

Mark only one oval.

- Yes
- No

Part 4: exposure to medicolegal problem (before or after college, inside or out side college at any time of your life)

59. Exposed to sexual harassment *

Mark only one oval.

- Yes
- No

60. Watch pornographic pictures and movies *

Mark only one oval.

- Yes
- No

61. Cheating in exams *

Mark only one oval.

- Yes
- No

62. Do you feel guilty if you have cheated in the exam

Mark only one oval.

- Yes
- No

63. Stole anything from your university or from your colleagues *

Mark only one oval.

- yes
- No

64. Do you smoke? *

Mark only one oval.

- Yes
- No

65. Have you performed any form of bullying to your colleagues? *

Mark only one oval.

- Yes
- No

66. Have you exposed to any form of bullying by your colleagues or professors? *

Mark only one oval.

- Yes
- No

67. Have you exposed to any form of black mailing? *

Mark only one oval.

- yes
- no

knowledge about the medicolegal problem

68. In case of illegal suspicion you must notify the police authority immediately through official procedure? *

Mark only one oval.

- Yes
- No
- Don't know

69. In the same context, should you notify the relatives about your suspicion prior to police notification? *

Mark only one oval.

- Yes
- No
- Don't know

70. Do you submit to any sort of pressure/stress preventing you from disclosing any criminal suspicion *

Mark only one oval.

- [ ] Yes
- [ ] No

This content is neither created nor endorsed by Google.

Google Forms
